# Assessing personality in San Joaquin kit fox in situ: efficacy of field-based experimental methods and implications for conservation management

**DOI:** 10.1007/s10164-017-0525-9

**Published:** 2017-09-12

**Authors:** Samantha Bremner-Harrison, Brian L. Cypher, Christine Van Horn Job, Stephen W. R. Harrison

**Affiliations:** 1 Endangered Species Recovery Program, California State University, Stanislaus, CA USA; 2 School of Animal, Rural and Environmental Sciences, Nottingham Trent University, Southwell, Nottinghamshire NG25 0HU UK

**Keywords:** Reintroduction, Boldness, Personality, Novel object test, San Joaquin kit fox, Conservation, In situ

## Abstract

Utilisation of animal personality has potential benefit for conservation management. Due to logistics of robust behavioural evaluation in situ, the majority of studies on wild animals involve taking animals into captivity for testing, potentially compromising results. Three in situ tests for evaluation of boldness in San Joaquin kit fox (*Vulpes macrotis mutica*) were developed (ENOT: extended novel object test; RNOT: rapid novel object test; TH: trap/handling test). Each test successfully identified variation in boldness within its target age class(es). The TH test was suitable for use across all age classes. Tests were assessed for in situ suitability and for quantity/quality of data yielded. ENOT was rated as requiring high levels of time, cost and labour with greater likelihood of failure. However, it was rated highly for data quantity/quality. The TH test was rated as requiring little time, labour and cost, but yielding lower quality data. RNOT was rated in the middle. Each test had merit and could be adapted to suit project or species constraints. We recommend field-based evaluation of personality, reducing removal of animals from the wild and facilitating routine incorporation of personality assessment into conservation projects.

**Electronic supplementary material:**

The online version of this article (doi:10.1007/s10164-017-0525-9) contains supplementary material, which is available to authorized users.

## Introduction

Study of animal personality has increased in popularity over recent decades, with theoretical and applied advances in fields such as behavioural ecology, animal welfare and conservation. Personality is defined as consistent behavioural responses expressed by individuals that are stable across time and/or contexts (Coleman and Wilson 1998; Sih et al. 2004, 2012). Personality studies relating to reproductive success (Carlstead et al. 1999; Mutzel et al. 2013), species reintroduction (Bremner-Harrison et al. 2004; Sinn et al. 2014) and individual susceptibility to disease (Boyer et al. 2010; Kortet et al. 2010) highlight the importance of predictable consistent individual responses to conservation efforts.

As a result, incorporating personality evaluation into conservation programmes has been recommended in empirical studies (e.g. Bremner-Harrison et al. 2004; McPhee and Silverman 2004) and theoretical/review papers (e.g. McDougall et al. 2006; Watters and Meehan 2007). In particular, it is suggested that the personality of an individual may serve to predict likelihood of survival or dispersal from a release site (Pinter-Wollman 2009; Bremner-Harrison et al. 2013). Conducting an assessment of personality at a captive-breeding facility can be implemented with limited labour/financial resource implications, particularly where validated protocols exist. However, assessment of wild, free-living animals is logistically more problematic, particularly if the species in question is unsuited for testing in a mobile field-testing station (such as the open-field test arena used by Martin and Réale 2008). Captive holding for behavioural assessment requires fiscal and human resources, both of which are often limited within conservation programmes. This generates a need for reliable in situ tests that are relatively quick and easy to perform under field conditions, are repeatable, have limited contact with the animal, and generate data that can be analysed within a timescale sufficient to enable incorporation of personality data into species or population decision-making processes.

Consequently, while personality assessment of individuals or groups of animals has become relatively wide-spread, differing methodologies are favoured according to situation. Watters and Powell (2011) provide an evaluation of the ratings, coding and experimental methods of assessment. While their review focusses on application in the captive context, pros and cons listed for experimental behaviour tests, e.g. the ability to draw out individual differences in behaviour versus difficulties in standardising experimental conditions, are applicable to wild-based studies.

A common approach to standardise experimental testing conditions in studies of wild animals living in situ typically involves a period of holding animals in captivity while behavioural assays are performed. Animals are then re-released for data collection on associated ecological or evolutionary parameters (e.g. Dingemanse et al. 2012). While this is advantageous in terms of standardising conditions for repeatable testing, there is a concern that results obtained in temporary captivity may not reflect results if the tests had been conducted in situ (Stratton 2015; Archard and Braithwaite 2010). Where animals do remain in the wild for behavioural testing, methods typically restrict movement of the animal for the full duration of the test, for example by physically restraining the animal (Réale et al. 2000), or placing the animal in a handling bag or open-field test arena (Armitage 1986; Brown et al. 2005; Martin and Réale 2008). Furthermore, experimental wild-based studies typically focus on birds (e.g. Gabriel and Black 2010), fish (Wilson et al. 1993) or reptiles (e.g. Carter et al. 2012). In situ experimental evaluations of personality of wild mammals are limited, and this is more so when searching the peer-reviewed published literature for assessment of carnivore personality (however see Dunston et al. 2016 for boldness assessment of lions via playback experiments).

Following assessment of the impact of personality on reintroduction success, Sinn et al. (2014) and Bremner-Harrison et al. (2004) recommend incorporating personality assessment into reintroduction strategies. Reintroduction and translocation programmes have shown a bias towards mammalian carnivore species (Breitenmoser et al. 2001), therefore for personality assessment to be reliably incorporated, robust personality tests suitable for carnivores are required. Previous ex situ personality assessment of release candidates demonstrated significant association between levels of boldness and post-reintroduction survival and dispersal (Bremner-Harrison et al. 2004; Sinn et al. 2014), indicating that boldness is a personality trait relevant to conservation efforts. Furthermore, boldness has been identified as having a key trade-off effect with survival and reproductive output (Smith and Blumstein 2008). Therefore, development of repeatable field-based tests focussed on assessing variation in boldness is recommended due to the likely impact of boldness on reintroduction success.

Experimental tests for measuring boldness in a free-living population of San Joaquin kit fox (*Vulpes macrotis mutica*) were developed and assessed to determine suitability for wild-based evaluation of personality. The San Joaquin kit fox historically occupied arid upland habitats throughout the San Joaquin Valley, California, USA. Former and current conversion of these habitats to agricultural, industrial and urban uses has resulted in profound habitat degradation, fragmentation and loss. As a result, the San Joaquin kit fox was listed as Federally Endangered in 1967 and California Threatened in 1973 (U.S. Fish and Wildlife Service 1998). A long-term recovery strategy for San Joaquin kit foxes recommends reintroduction of foxes to recovered habitat, sourcing foxes from existing populations where removal will not negatively impact the source population (U.S. Fish and Wildlife Service 1998). The City of Bakersfield within the San Joaquin Valley has a sustainable population of kit foxes that may constitute a potential source of reintroduction candidates. However, due to concerns regarding behavioural suitability of an urban fox population for reintroduction compared with rural foxes (Bremner-Harrison and Cypher 2007), a study of personality was undertaken, necessitating the development of appropriate testing procedures.

To test as wide a range of the population as possible, three tests were developed. Each test type was targeted to assess one or more age classes of foxes, and was suitable for the life-history constraints observed within the particular age class, such as movements, or body size. Tests varied in complexity, repeatability, duration and animal contact time. The aim of the study was to determine whether robust measures of personality for an endangered mesocarnivore could be found that were easy to perform in the field. Test objectives were to reliably assess boldness in situ, however an additional conservation aim was that individuals assessed could then be monitored in the field to obtain survival, movement and reproductive data. Therefore, in addition to the test itself being appropriate, how the test fitted into the wider conservation programme was taken into consideration. Subsequently, following execution of the tests, fieldworkers rated each test on factors relating to efficacy.

## Materials and methods

### Study area

The study took place within the City of Bakersfield, which is located in Kern County at the southern end of the San Joaquin Valley, California, USA. The City of Bakersfield covers approximately 300 km^2^ with the full metropolitan area covering 580 km^2^ (Bremner-Harrison and Cypher 2011). Bakersfield has a large self-sustaining population of kit foxes that appear to have successfully adapted to the urban environment, modifying their diet to include anthropogenic food items and reproducing within short distances of human businesses and residences (Cypher 2010).

### Boldness tests

Three assessments of boldness were developed suited to taxon-specific restrictions and field conditions to determine in situ boldness levels: extended novel object test (ENOT), rapid novel object test (RNOT) and trap/handling test (TH). A summary of the test type, focal group, number and types of observation, and sampling methods is provided in Table 1. Due to differing times spent visible at dens and differing frequency of den location changes across age classes, tests were developed to assess age classes independently.

**Table 1:** Characteristics of three behavioural tests used to assess boldness in San Joaquin kit foxes (*Vulpes macrotis mutica*) within the City of Bakersfield, California

Test ID	Test type	Focal group^a^	ID method	No. of observation periods	Sampling method
Extended novel object test (ENOT)	Experimental (response to novelty) + non-stimulus behavioural coding (Watters & Powell 2011)	Pups (<1 year old)	Dye mark	4 × 1 h 2× stimulus 2× coding	Instantaneous scan sampling at 1-min intervals (Martin and Bateson 1993)
Rapid novel object test (RNOT)	Experimental (response to novelty)	Juveniles (>1 year old, <2 year old) Adults (>2 years old)	Radio collar	1 × 1 h 1× stimulus	Continuous focal sampling (Martin and Bateson 1993)
Trap/handling test (TH)	Experimental: response to trapping and handling	All age classes	Ear-tagged as part of trapping process	Opportunistic as trapped	Binary: yes/no occurrence of behaviour

^a^Tests were developed to assess different age classes, with a subset of individuals assessed across all three tests as they reached the appropriate age class over time

### Pre-test trapping and handling

Foxes were trapped and either individually dye-marked (ENOT) or radio-collared (RNOT) prior to behavioural assessment. The trapping process is described in full in Cypher et al. (2009). In brief, adult and pup kit foxes were captured in wire-mesh box live traps measuring 38 × 38 × 107 cm^3^ (Tomahawk, Wisconsin, USA) that were set at dusk, covered with a tarpaulin to protect foxes from inclement weather and sun, and baited with a variety of food items (hot dogs, hard-boiled eggs, dry and wet cat food and bacon). Water was not provided in traps, as kit foxes source their water from prey items (Golightly and Ohmart 1984). To minimise risk of tooth injuries, each trap contained two rope chew toys, with one attached to the bottom of the door at either end of the trap. Traps were checked at dawn; any trap not containing a fox was collapsed and removed to prevent entry by any other animals during daylight hours.

Captured foxes were coaxed from the trap into a handling bag (75 × 75 cm^2^); the bag restrained the fox and covered its eyes during the handling process. Manual restraint removed the need for chemical immobilisation and associated risks. Foxes were weighed, sexed, assessed for reproductive condition, ear-tagged, aged and checked for injuries. Foxes above a minimum weight of 1.16 kg were fitted with a radio-telemetry collar weighing 35 g (Advanced Telemetry Systems, Isanti, Minnesota, USA) for RNOT, or with a dye mark for ENOT. Once handling was completed, foxes were released at the site of capture. All handling was concluded within 1–2 h of sunrise. Kit fox trapping and handling techniques were identical across each test method other than the addition of dye marking or fitting of radio-collars where appropriate. Trapping took place between 1 May and 15 January each year from 2005 to 2009. All handlers were fully trained and signed off as competent in fox trapping and handling as per permit requirements.

### Extended novel object test

The ENOT was originally developed for captive foxes of all age classes (see Bremner-Harrison et al. 2004 for details). However, this test comprised viewing individuals for four sessions, each lasting 1 h (Table 1), which required individuals to remain in one location for the duration of the observation period. Pups remain at the natal den site while active, whereas adults and juveniles are less predictable, as at the start of the activity period they will leave the daytime resting den to hunt as required and are unpredictable in their movements. Therefore, the in situ ENOT focussed on pups only. The protocol comprised trapping and radio-collaring adults in winter, then tracking the adults through the breeding season to locate natal dens in the spring for assessment of pups. Once natal dens were confirmed, pups were trapped as above, individually marked using a non-toxic dye (Nyanzol-D, Belmar Inc., North Andover, Massach, USA) and released back into their natal den.

A further modification to the test from Bremner-Harrison et al. (2004) was the change from four different novel object stimuli observations to a combination of coding and novel object stimuli observations, in order to obtain repeatable non-manipulated data and ‘novel beneficial stimulus’ and ‘novel threatening stimulus’ personality data. Thus, ENOT comprised four observation sessions: 2 × novel stimuli observations (1 × potentially beneficial and 1 × potentially threatening), and 2 non-stimuli coding observations. The duration of each observation was 60 min, giving a total of 240 min of observation for the ENOT.

**Fig. 1:**
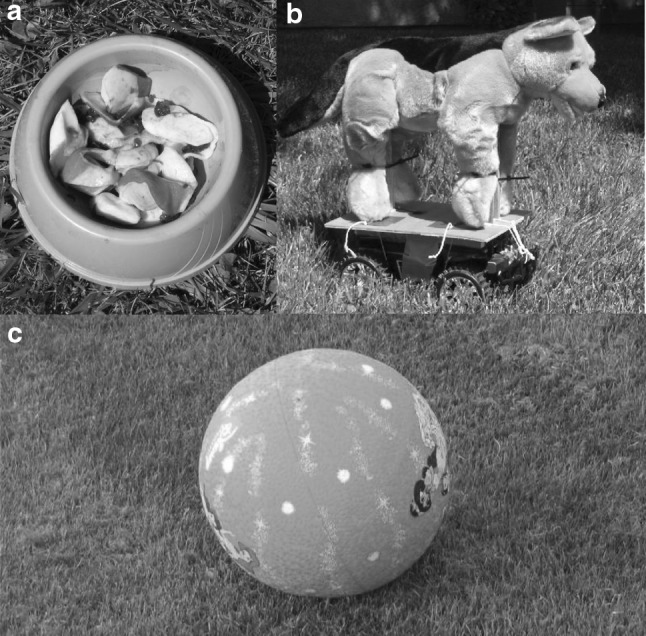
Novel objects presented to San Joaquin kit fox (*Vulpes macrotis mutica*) for assessment of boldness in Bakersfield, California. Objects (**a**) as a potentially beneficial stimulus and (**b**) as a potentially threatening stimulus were presented during the extended novel object test, and object (**c**) during the rapid novel object test

The potentially beneficial stimulus (Fig. 1a) was a novel food source not previously encountered by the foxes, presented in a pet food bowl, consisting of imitation crab meat (Krab Sticks, Berelson Co., California, USA), Mouse-Special Bait—a commercially available trapping bait (R & M Lures, Iowa, USA) and Canine Call—a commercially available trapping lure (The Snare Shop, Iowa, USA). The potentially threatening stimulus (Fig. 1b) was designed to assess the behavioural response to a potential risk. A plush toy dog (Toys R Us, USA) doused in coyote urine (The Snare Shop, Iowa, USA) was mounted onto the base of a modified remote-controlled toy vehicle (dimensions including base: *L* 50 cm × *H* 55 cm). An internal CD player and speakers (Sony, USA) played a series of coyote (*Canis latran*) howls and a coyote–grey fox (*Urocyon cinereoargenteus*) fight interaction. Whilst coyotes are the main source of mortality for kit foxes in rural habitats (Cypher and Spencer 1998), they were rarely observed at the urban study site and were considered unfamiliar to both pups and adults.

Observation set-up commenced 1 h prior to sunset, to ensure that the start of the observation concurred with the normal crepuscular/nocturnal kit fox activity periods for emergence from the natal den (Morrell 1971). As San Joaquin kit foxes occasionally move natal den sites, prior to the start of each observation, the radio-collared adult female was tracked to confirm den location. The ENOT took place over a minimum of 4 days, commencing at >36 h after capture. A minimum interval of 24 h was applied between the four observation periods. The observation periods were conducted in random order (stimulus plus type and non-stimulus). Each of the two novel stimuli were presented once. The novel stimulus was placed at the den site and behaviour observed using 12 × 50 binoculars (Ranger Edition, Eagle Optics, Wisconsin, USA), and recorded via digital voice recorder (Mio168 DigiWalker, Mio Technology, Taiwan). Observations were also filmed (Sony Handycam DCR-HC46, Sony, USA) for reference. Distance of the object from the den entrances varied according to the number of entrances to the natal den and the topography of the ground; typically, the object was located between 0.5 and 2 m from the centre of the den site. The observer distance varied depending on topography, including location of buildings, but was a minimum of 80 m. Level of concealment was also site specific; at dens located in areas of regular human activity such as university campuses and high schools, the observer was located at a site where human presence was frequent, such as a bench outside a building, or on stadium bleachers. For dens in less populated areas, such as on undeveloped sites or water-collection sumps, the observer was partially concealed. A pre-observation habituation period determined levels and placement of concealment for each den site.

The behaviour of each pup was recorded using a modified ethogram previously developed for captive swift fox (Bremner-Harrison et al. 2004) (Table S1). Using previously developed methods for scoring swift fox behaviour (Bremner 2002; Bremner-Harrison et al. 2004), behavioural activities within the ethogram were scored as extremely bold = 3, bold = 2, shy = 1, and extremely shy = −1 (Table 2), and the number of occurrences of each scored behaviour summed to produce an overall boldness score for the observation session. Boldness was scored for each individual for each observation session, and summed to give an overall score across the four sessions. Higher scores represent higher levels of boldness.

**Table 2:** Categorisation of scored behaviour in the extended novel object test (ENOT) assessing boldness in San Joaquin kit fox (*Vulpes macrotis mutica*) in Bakersfield, California

Extremely bold		Bold		Shy		Extremely shy	
Beh. coding	Novel object	Beh. coding	Novel object	Beh. coding	Novel object	Beh. coding	Novel object
Left den site	Investigating	Resting relaxed	Resting relaxed	Resting alert	Resting alert	In den	In den
	Bold approach (object)	Stretching	Stretching	Sniffing	Sniffing	Warning bark	Warning bark
	Pouncing on object	Rolling	Rolling	Hesitant approach (conspecific)	Hesitant approach (object)		
	Pouncing on conspecific	Investigating	Bold approach (conspecific)	Fleeing	Hesitant approach (conspecific)		
	Fighting over object	Bold approach (conspecific)	Chasing conspecific	Fleeing conspecific	Fleeing		
	Play chase	Chasing conspecific	Following conspecific	Food offering	Fleeing conspecific		
	Play flee	Following conspecific	Stalking	Grooming conspecific	Watching conspecific		
	Play fight	Stalking	Discipline	Watching conspecific	Watching observer		
	Play stalk	Pouncing on conspecific	Eating	Watching observer	Watching people		
	Playing with object	Discipline	Food gathering	Watching people			
	Left den site	Play chase	Food offering				
		Play flee	Food beg				
		Play fight	Caching				
		Play stalk	Unearthing food				
		Playing with object	Hunting				
		Eating	Marking				
		Food gathering	Grooming self				
		Food beg	Grooming conspecific				
		Caching	Greeting conspecific				
		Unearthing food	Food carrying				
		Hunting					
		Grooming self					
		Greeting conspecific					
		Food carrying					

Behavioural categories scored as: extremely bold = 3, bold = 2, shy = 1, extremely shy = −1

Fifteen adult kit foxes were trapped and collared between 1 October 2005 and 15 January 2006 and tracked weekly to their day-time resting locations to determine the presence of natal dens. Between May and July 2006, 21 pups (11M:10F) were captured over 176 trap nights across five natal dens, with 15 repeat captures (7M:8F). The ENOT was conducted from 15 May to 3 July 2006 on 24 pups at the five natal dens, and boldness scores calculated for each individual.

### Rapid novel object test

The RNOT comprised focal observations of radio-collared adult and juvenile foxes. Thirty-six hours post-trapping/collaring, foxes were tracked to their day-time resting location and a novel object placed at the den entrance, in such a position where the fox would be required to almost fully emerge from the den prior to seeing the object (Fig. 1c). The novel object, a ball approximately 40 cm in diameter (Toys R Us, USA), was considered neither potentially beneficial nor threatening. On emerging from the den, the fox’s behaviour was recorded using an ethogram modified from ENOT (Table S2), again using 12 × 50 binoculars, the digital voice recorder and digital video recorder. If a fox did not emerge during the observation period, and the radio-telemetry signal did not indicate movement by the fox towards the den entrance from within the den, the novel stimulus was removed and the observation repeated on a subsequent day.

As data were collected via continuous sampling, duration in seconds of each observed behaviour was recorded and per cent duration for each type of behaviour scored according to boldness level. Summed duration of bolder behaviour was scored as 2 and of shyer behaviour as −1 (Table 3). Bold and shy scores were summed to give an overall boldness score along a shy–bold continuum for each fox observed. Again, higher scores represent foxes that performed greater duration of bold-type behaviour. ‘Not emerged from den’, ‘Locomotion’ and ‘Left den site’ were excluded, as it was not possible to ascertain whether these categories were motivated by the presence of the stimulus or variables unconnected to the novel object test, for example the motivation to begin hunting. Due to some individuals leaving the den site during the observation period, foxes were not visible for equal time periods within observations, thus data were transformed into proportional data for the time visible.

**Table 3:** Behaviours scored as bold (=2) and shy (=−1) in the rapid novel object test for assessing boldness levels in adult and juvenile San Joaquin kit fox (*Vulpes macrotis mutica*)

Bold behaviour	Shy behaviour
Investigating novel object	Observing novel object
Investigating general	Vigilant/resting alert
Resting relaxed	Retreat
Approach	Back in den
Grooming	

Twenty-seven adult and juvenile foxes (13A:14J) were trapped and radio-collared from 2006 to 2009. The RNOT was conducted between 19 December 2006 and 5 May 2009 (10M:17F).

### Trapping/handling test

Each time a fox was trapped, behaviour was recorded on a ‘yes’ or ‘no’ binary basis (yes = 1, no = 0) regarding whether particular behaviours were observed during the capture process (Table S3). Behaviours were classified as shy or bold, and given a weighting according to category (shy = −1, bold = 1). The data were transcribed as numerical values with a zero if a behaviour was not performed, and either 1 or −1 for performance of a bold or shy behaviour. A boldness score per trapping event was calculated by summing the occurrences of shy and bold behaviour to give a boldness value. As there was a likelihood that binary occurrences of bold and shy behaviour may cancel one another out for some foxes, data were transformed by adding 0.5 to each boldness score and the resultant value was divided by the sum of shy and bold behaviors performed by each individual (Veber 2009). Mean boldness scores across captures were used for analysis to account for variation in handler experience.

Eighty-seven adult, juvenile and pup kit foxes were trapped and handled between July 2006 and June 2009 (52M:35F; 33A:6J:48P). Between one and four TH behavioural assessments per individual were performed depending on number of times captured ($\bar{X} \pm {\text{SE}} =$ 1.46 ± 0.08, *n* = 87).

### Efficacy of field-based effort

Measurement of field-based effort was utilised as a means of assessing (1) whether each test was appropriate in terms of suitability for use with free-living animals and (2) the quality and quantity of data obtained. To assess time and sample size efficiency, a ‘data return rate’ was calculated by dividing the number of individuals assessed by the number of weeks taken to complete the behavioural evaluation. Researchers involved in all three tests (ENOT, RNOT and TH) evaluated each test in terms of the following: duration, labour, repeatability, results obtained, quantity and quality of data, expense, and potential for the experiment to fail. Categories were rated on a Likert scale from 1 to 5 (S4). The aim was not to compare between test types, but to evaluate the efficacy of each test in its own right for use in situ as a measure of boldness and for assimilation with subsequent monitoring goals.

### Data analysis

Results from the three measures of boldness were evaluated to (1) determine whether each method identified variability in boldness amongst the individuals tested (one-group variance test), (2) determine, where appropriate, whether each method identified individual consistency in behaviour (Kendall’s coefficient of concordance; Siegel 1956), (3) provide a measure of efficacy in terms of ease of execution, duration, results obtained and reliability of method and (4) determine whether individuals measured across more than one method showed similar levels of boldness. Data were analysed using SPSS version 19.0.

## Results

### Variation in boldness between individuals

All three tests identified variability in boldness within the sample sets (Table 4, Fig. S5). Data were analysed to determine whether the tests detected variation between individuals, sex, or where applicable, age. Of the three tests, ENOT was found to extract the greatest levels of variability within the sample set, with significantly higher variance than the RNOT or TH test [Table 4; *F*_(2)_ = 55.2, *P* < 0.0001; modified Levene’s test (Hines and O’Hara Hines 2000)].

**Table 4:** Descriptive statistics and variance of boldness scores obtained from the ENOT, RNOT and TH tests for assessing boldness in San Joaquin kit fox (*Vulpes macrotis mutica*) in Bakersfield, California

Test	*N*	Mean ± SD	Range	Variance	Difference in variance from hypothesized value of 1	Duration (weeks)	Data return
ENOT	24	−40.5 ± 128.9	513.0	16,612.6	*χ* ^2^ = 382,089.9, *df* = 23, *P* < 0.0001	41	0.59
RNOT	27	−19.5 ± 76.1	245.6	5788.9	*χ* ^2^ = 150,512.9, *df* = 26, *P* < 0.0001	103	0.26
TH	87	0.4 ± 0.3	1.67	0.1	*χ* ^2^ = 9.0, *df* = 86, *P* < 0.0001	150	0.58

Between-den differences were assessed for the ENOT (*F*_4_ = 3.097, *P* < 0.05, ANOVA), with pups from den 3 significantly bolder than pups from den 1 or den 5 (*P* < 0.005; *P* < 0.005, post hoc Fisher’s PLSD). The ENOT also detected within-litter differences across all dens (den 1: *χ* ^2^ = 27,892.8, *df* = 3, *P* < 0.0001; den 2: *χ* ^2^ = 86,063.4, *df* = 6, *P* < 0.0001; den 3: *χ* ^2^ = 113,874.0, *df* = 4, *P* < 0.0001; den 4: *χ* ^2^ = 4140.5, *df* = 1, *P* < 0.0001; den 5: *χ* ^2^ = 8823.3, *df* = 5, *P* < 0.0001). No gender- or age-related variation in boldness scores was observed.

### Repeatability

The ENOT test comprising four observation periods was conducted once per den, therefore repeatability of the overall test could not be assessed, however consistency of response was assessed across the four observation periods. Individual boldness across the four observations was consistent (*W* = 0.4, *X*_23_^2^ = 38.6, *P* < 0.05; Kendall’s coefficient of concordance, corrected for tied ranks, Siegel 1956), reflecting repeatability of the measure. Significantly higher boldness scores were displayed in the presence of the potentially beneficial stimulus than in the presence of the potentially threatening stimulus (*t*_23_ = 3.7, *P* < 0.001). Therefore, a fox that was ranked highly for boldness in one observation ranked highly across all four observations despite a reduction in boldness shown in response to variation in stimulus type.

The TH test was variable in the number of repeats per individual, with 1–4 assessments per individual ($\bar{X} \pm {\text{SE}} =$ 1.47 ± 0.08, *N* = 87). Analyses of differences between repeated scores per individual were non-significant, indicating within-individual consistency of scores. RNOT was conducted once per fox, therefore no measure of repeatability was possible.

Eleven individuals had boldness assessed using all three tests as they progressed through age classes. Boldness at the individual level was consistent across the three tests (*W* = 0.4, *X*_10_^2^ = 15.0, *P* < 0.05; Kendall’s coefficient of concordance, corrected for tied ranks); i.e. the individual boldness ranking of each of the 11 foxes remained the same regardless of which test was used.

### Duration and ‘return’ of each test

The duration from the initial locating of foxes to the final data collection varied across the three tests. ENOT duration comprised 41 weeks from date of first trapping and collaring of an adult fox (for locating natal dens) to final novel object boldness test at a natal den site. RNOT took place over 103 weeks, from date of first collaring to final novel object boldness test. The TH behavioural assessment took place over 150 weeks, spanning the duration of both ENOT and RNOT. When considering the number of individuals assessed relative to the duration of the behavioural test, the ‘data return rate’ (individuals/week) was 0.59 for ENOT, 0.26 for RNOT and 0.60 for TH.

### Efficacy of the three boldness tests

The field researchers (*N* = 3) who conducted the three tests rated them for efficacy of assessing boldness in situ. Rating was done for each test independently, rather than a comparative rating across tests. ENOT was rated as having long duration, high labour requirements, high likelihood of failure and high expense (Fig. S5). However, it was rated as having the capacity to yield high quantities of data of the highest quality. The TH test was rated as having short duration, requiring low levels of labour, low expense and having low risk of failure. However, while the TH test was rated as having the capacity to produce large quantities of data, this was rated at a low quality level. The RNOT was rated at the mid-point of the scale (see Table S4 for ratings variables and scales) for duration, labour, data quantity and quality, expense and risk of failure. The RNOT was rated highly for the capacity for repeatability of tests.

## Discussion

Confidence in in situ testing methods is imperative for incorporating personality evaluation into future conservation management. Our results indicate that each test type was a viable means of assessing boldness in one or more age classes of a free-living population of mammals whereby a measure of boldness was obtained and variation in boldness levels identified. Levels of variability detected differed across tests, with significantly more variation identified in the lengthier and more labour-intensive tests. However, this increased variation may also have been a function of behaviours being scored at more levels of boldness for ENOT and RNOT compared with TH. Increasing the number of behaviours assessed, and rating individuals on a scale would potentially increase the variation detected by the TH test.

ENOT and TH each demonstrated consistency of boldness for individuals across multiple assessments, confirming the repeatability of the test procedures. While RNOT could not be assessed for repeatability due to presentation of only one stimulus, significant concordance was found between the individual boldness levels of the 11 individuals assessed using all three tests, demonstrating consistency in boldness scores. This consistency across the three methods of determining boldness suggests that the RNOT is capable of providing a reliable measure of boldness, providing confidence in the test. However, should the RNOT test be used on its own, we recommend repeat assessment of each individual to provide a means of confirming repeatability of scores. Given the findings presented, we suggest that all three methods offer a robust means of assessing boldness in free-living mammals across a range of age classes. Furthermore, whilst in this particular study the focus was on assessing individuals within the context of the shy–bold continuum given its relevance to reintroduction (Sinn et al. 2014), each testing method provides the means to assess a wider range of personality domains and across different contexts.

Researcher ratings of each method provided a means of evaluating efficacy when used in the field. Overall, field researcher ratings indicated that, while TH was the easiest of the three methods to conduct in the field in terms of duration, labour, expense and data quantity, ENOT and RNOT generated data of higher quality. However, whilst both ENOT and RNOT yielded high-quality data, ENOT was considered as having higher likelihood of failure than RNOT. Furthermore, ENOT is limited in its application, as in this instance it required individuals to be spatially limited to one location as they were too young for radio-collaring. However, as suggested above, the RNOT could be extended to encompass multiple assessments of individuals using a range of stimuli to incorporate both repeatability and context specificity. Thus, ultimate viability or choice of method employed will depend on the overall goal, duration and resources of the project.

Despite being ranked as generating high quality of boldness data, due to the intensity of the method and likelihood of failure, we do not consider ENOT to be as well suited as RNOT or TH if the overall project goal is to select release candidates for a reintroduction programme. The ENOT methodology was multi-faceted and labour intensive, incorporating both the breeding season and pup-rearing seasons for the species. In addition, the method excluded the opportunity for comparison across age classes, as unlike adults and juveniles, only pups reliably remained at one site for the duration of four 1-h observation periods. While parent foxes were present in the den prior to the set-up for an observation, during the actual observation period presence of parents was highly variable as they would return with prey items and then leave again. Therefore, in this instance, adult boldness was not assessed, as motivating behaviour was likely to have been the need to supply food for the pups rather than presence of a novel object. This restricted the ENOT in situ to only providing data on pups, whereas in captivity it can incorporate both pups and adults.

We consider RNOT to be well suited for in situ personality testing, particularly if the project goal is to identify source candidates for a founder population. The most notable benefit of this method is that individuals were trapped and collared prior to the observations taking place. Consequently, individuals can be precisely located for testing and the method facilitates subsequent tracking and re-capture of an individual identified as a suitable candidate for relocation. While in this instance the method was not suitable for pups due to the requirement for minimum weight gains to be reached for collaring, it can be used effectively on juveniles and adults, providing a means of testing a large proportion of the population. Furthermore, during the actual testing period, the tests can be conducted ‘hands-off’ unlike the TH test, which may produce more reliable results (Archard and Braithwaite 2010). Programmes where tagging is not constrained by the need to use a collar may be able to implement this test across all classes.

Although RNOT yielded a lower ‘data return rate’ than ENOT, in actuality RNOT was cheaper and faster to conduct due to its reduced resource requirements. ENOT was by necessity conducted in a short time window from when pups first emerged to the time of dispersal from the natal den site. Therefore, the behavioural study was intensive with field personnel focussed entirely on ENOT data collection during that time period. RNOT behavioural observations were conducted in conjunction with other personnel duties, which is more representative of data collection within an ongoing conservation project versus an academic study. It is unlikely that staff within a conservation programme would be able to devote 100 % of their time to collecting and analysing behavioural data, therefore a method of assessment that fits around other duties is beneficial. If, however, one member of staff was able to focus on RNOT data collection, an increased data return rate could be achieved. Thus, with factors such as labour intensity, time for completion and financial constraints being important considerations within a conservation programme, the methods employed in RNOT would appear favourable for in situ conservation application.

The TH method of obtaining data was not labour intensive and provided the largest sample size, as it was appropriate for use across all age classes. Data return rates were equal to those of ENOT; furthermore, the speed of execution enabled boldness data to be obtained from foxes trapped for other monitoring projects taking place concurrently to the behavioural study. Ease of application would allow this method to be used for projects that routinely trap and handle individuals, as exemplified by Réale et al. (2000). This method of collecting boldness data could be particularly useful during follow-up monitoring of a reintroduced population in order to determine the most suitable behaviour types for additional founders. However, the TH data were collected whilst animals were briefly restrained rather than ‘hands-off’, and therefore assessed whilst potentially in a stressed state (Teixeira et al. 2007). Such conditions may compromise temperament assessment (Archard and Braithwaite 2010) and may reduce the reliability of the boldness measure. However, the significant associations for boldness scores for the 11 individuals assessed across the three tests impart confidence in the scores obtained from the TH method.

The TH test detected low levels of variability within the population. This test may be improved by altering the method of recording boldness to assess behaviours on a Likert or visual analogue scale rather than the binary presence/absence method used. Furthermore, the TH test does not allow for the assessment of context-specific boldness. Comparison of boldness in response to the potentially beneficial stimulus and the potentially threatening stimulus used in ENOT demonstrated significantly lower levels of bold behaviour in presence of the fake predator. Context-dependent variation would not be able to be assessed using the TH method where trapping and handling methods are necessarily kept constant, but could be incorporated into RNOT with an increase in observation periods and change test context. We would recommend that the TH test be used where trapping/handling are occurring anyway as an opportunistic routine method of data collection.

All three methods tested here required the trapping of individuals at some stage of the process, which inherently results in bias towards bolder individuals (Carter et al. 2012; Biro and Dingemanse 2009), potentially excluding individuals from the shyer end of the behavioural spectrum (Wilson et al. 1993). However, use of focussed trapping methods such as ‘hood-trapping’ (funnelling from den entrances into traps) would increase capture of animals that avoid traps, thus reducing bias. Once trapping was complete, ENOT and RNOT were conducted with minimal disturbance to the animals. The outcomes of these methods were considered representative of novelty and risk in the wild, without the confounding effects of removal from the wild during testing.

In conclusion, of the three methods evaluated for assessing personality in the field, RNOT was deemed the most appropriate to in situ assessment. RNOT generated robust data and pre-collaring allows for repeat assessment, tracking individuals for capture and translocation, or for gathering further life-history data. Furthermore, RNOT provides the means of modification to the novel object assessments, allowing for context-specific testing. The TH test is an excellent measure for behavioural analysis within a study where resources are restricted, or projects that routinely carry out trapping for other purposes, but we recommend using a more informative scale to provide greater definition between individuals. Since development of the three tests, they have been successfully utilised on a range of species, including the in situ TH test on wood mice (*Apodemus sylvaticus*) (Stratton 2015), in situ RNOT and TH test on swift fox (*Vulpes velox*) (Veber, 2009) and the ‘hands-off’ behavioural coding aspect of ENOT on ring-tailed lemurs in a free-ranging/walk-through ex situ exhibit (Smith 2015).

With growing evidence of the impact of personality on species survival and fitness (Dingemanse et al. 2012; Smith and Blumstein 2008; Kortet et al. 2010; Mutzel et al. 2013), there is a clear role for personality research in real-world conservation strategies. Despite the growth of reintroduction biology as a discipline within conservation biology (Seddon et al. 2007), the study of personality within this field is limited. However, previous research has highlighted the potential benefits of including behavioural selection criteria when creating founder groups for species reintroduction (Bremner-Harrison et al. 2004). Thus, the ability to assess the personality of wild, free-living animals across a range of contexts for a variety of species by means of an in situ method that is logistically feasible and reliable in its delivery of robust data is imperative. The results presented here provide an advancement of applied techniques relevant to conservation.

## Electronic supplementary material

Below is the link to the electronic supplementary material.

Supplementary material 1 (DOCX 16 kb)

Supplementary material 2 (DOCX 13 kb)

Supplementary material 3 (DOCX 13 kb)

Supplementary material 4 (DOCX 18 kb)

Supplementary material 5 (DOCX 22 kb)

Supplementary material 6 (DOCX 23 kb)
