# Supplementary material for: Assessing personality in San Joaquin kit fox in situ: efficacy of field-based experimental methods and implications for conservation management
Source: J Ethol. 2017 Sep 12;36(1):23–33. doi: 10.1007/s10164-017-0525-9 (PMC5746588; doi:10.1007/s10164-017-0525-9)
Supplement: Supplementary file 1 — Supplementary material 1 (DOCX 16 kb) [file 10164_2017_525_MOESM1_ESM.docx]

**Table S1:** Behavioural ethogram used for assessing boldness of San Joaquin kit fox pups using the Extended Novel Object Test (ENOT).^a^ denotes behaviours used for behavioural coding, and ^b^ for stimulus observations

| Behaviour | Behavioural definition |
| --- | --- |
| 1. In den^a^ | below ground in the den |
| 2. Resting relaxed^a^ | lying or sitting in relaxed posture/asleep, ears lowered, eyes may be closed |
| 3. Resting alert^a^ | lying, sitting or standing with ears erect and eyes open |
| 4. Stretching^a^ | elongating limbs with a bout of yawning |
| 5. Rolling^a^ | rubbing face and body on ground or object |
| 6. Walking | slowest gait of locomotion |
| 7. Trotting | steady pace faster than walk, lift diagonal pairs of legs |
| 8. Loping | slow bouncy run |
| 9. Running | fastest pace of locomotion |
| 10. Jumping | leaping either into the air or on an object |
| 11. Climbing | prolonged effort to climb up an object |
| 12. Sniffing^a^ | sniffing at the air, nose up |
| 13. Investigating^a^ | walking, running or standing sniffing at ground or object in enclosure |
| 14. Bold approach (object) | direct approach towards novel stimulus, ears erect |
| 15. Bold approach (conspecific) ^a^ | direct approach towards conspecific, ears erect |
| 16. Hesitant approach (object) | slow approach towards novel stimuli with frequent retreats and advances, ears and body usually lowered |
| 17. Hesitant approach (conspecific) ^a^ | slow approach towards conspecific with frequent retreats and advances, ears and body usually lowered |
| 18. Chasing conspecific^a^ | chasing a conspecific not in play, often away from a novel stimulus or food item |
| 19. Fleeing | run towards den or away from object, often in response to a warning bark |
| 20. Fleeing conspecific^a^ | moving quickly away from conspecific |
| 21. Following conspecific^a^ | moving slowly behind a conspecific, not chasing |
| 22. Stalking^a^ | approaching an object/prey item in a crouched position |
| 23. Pouncing on object | leaping onto an object using forelegs to land, often occurs after stalking |
| 24. Pouncing on conspecific^a^ | leaping onto conspecific, often occurs during an existing play bout, or as an invitation to play |
| 25. Fighting | aggressive interaction between conspecifics |
| 26. Fighting over object | aggressive interaction as a result of competition over object |
| 27. Discipline^a^ | snapping or growling at a conspecific, may knock them to the ground and stand over them. |
| 28. Submission | directed towards conspecific, lowered posture, ears flattened, often wagging tail |
| 29. Play chase^a^ | running, chasing alone or with other conspecifics, often alternate role of pursuer |
| 30. Play flee^a^ | running away from a conspecific or object, ears more erect than Fleeing. often alternate roles |
| 31. Play fight^a^ | wrestling, tumbling, biting and jumping with a conspecific |
| 32. Play stalk^a^ | slow approach to conspecific with body held low to ground, occurs within a play bout |
| 33. Playing with object^a^ | biting, tossing in the air, or jumping with an object, may be the novel stimulus |
| 34. Digging | using front paws to make holes |
| 35. Eating^a^ | all masticatory behaviors associated with food |
| 36. Drinking | intake of liquid |
| 37. Food gathering^a^ | collecting and carrying items of food in the mouth |
| 38. Food offering | presenting a food item to a conspecific |
| 39. Food beg^a^ | position of mouth and nose close to mouth of conspecific whilst wagging tail |
| 40. Caching^a^ | storing food item, usually in a small hole, and covering with debris |
| 41. Unearthing food^a^ | retrieving a previously cached food item |
| 42. Hunting^a^ | predatory behavior towards prey item, including stalking and jumping on/catching |
| 43. Defecating | discharge faeces or urine from body |
| 44. Scent marking | scent marking, either by depositing minimal amount of urine, scat, or rubbing body on a prominent object |
| 45. Grooming (self) ^a^ | biting, licking, nibbling or scratching at own body |
| 46. Grooming (conspecific) ^a^ | biting, licking, nibbling or scratching at a conspecific |
| 47. Greeting conspecific^a^ | ears back, head low, tail wagging |
| 48. Watching (conspecific) ^a^ | observing another fox within the enclosure |
| 49. Watching (observer) ^a^ | looking at the observer who is collecting data |
| 50. Pup carrying | adult moving pup to another area by scruff of the neck |
| 51. Warning bark^a^ | short loud bark, usually emitted by an adult at perceived danger |
| 52. Suckling | pup feeding by sucking at mother’s teat |
| 53. Entering den | moving out of sight into a den hole |
| 54. Leaving den | coming into view from a den hole |
| 55. Following person | moving at a distance behind a person |
| 56. Watching person^a^ | watching person within the enclosure |
| 57. Vomiting | regurgitation of food |
| 58. Left den site^a^ | is not present or is leaving from den site area (‘away’ when fox can no longer see the den) |
| 59. Arriving den site | returning to den site area after a period away |
| 60. Out of sight | fox is not visible |
| 61. Location unknown | unknown whether fox is in den or away from den |
| 62. Play bow | front elbows on ground, head lowered, rear in air, tail wagging |
| 63. Food carrying^a^ | bringing food into the den site or moving it around within den site |
