# Supplementary material for: Assessing personality in San Joaquin kit fox in situ: efficacy of field-based experimental methods and implications for conservation management
Source: J Ethol. 2017 Sep 12;36(1):23–33. doi: 10.1007/s10164-017-0525-9 (PMC5746588; doi:10.1007/s10164-017-0525-9)
Supplement: Supplementary file 2 — Supplementary material 2 (DOCX 13 kb) [file 10164_2017_525_MOESM2_ESM.docx]

**Table S2:** Behavioural ethogram used for assessing boldness of juvenile and adult San Joaquin kit fox using the Rapid Novel Object Test RNOT.

| **Behaviour** | **Behavioural definition** |
| --- | --- |
| Not emerged from den | Below ground in the daytime resting den |
| Observe novel object | Watching the novel object |
| Investigating novel object | Sniffing or pawing at the novel stimulus |
| Investigating general | Sniffing or pawing at an item or area other than the novel stimulus |
| Vigilant/Resting alert | Lying, sitting or standing with ears erect and eyes open |
| Resting relaxed | Lying or sitting in relaxed posture/asleep, ears lowered, eyes may be closed |
| Approach | Moving towards the novel stimulus |
| Retreat | Moving away from the novel stimulus |
| Out of sight | Fox is above ground but cannot be seen |
| Grooming | Biting, licking, nibbling or scratching at fur |
| Back In Den | Fox has gone back below ground |
| Locomotion | Moving around the den area at either a walk, trot, lope or run |
| Left Den Site | No longer present at the den site |
