# Supplementary material for: Assessing personality in San Joaquin kit fox in situ: efficacy of field-based experimental methods and implications for conservation management
Source: J Ethol. 2017 Sep 12;36(1):23–33. doi: 10.1007/s10164-017-0525-9 (PMC5746588; doi:10.1007/s10164-017-0525-9)
Supplement: Supplementary file 3 — Supplementary material 3 (DOCX 13 kb) [file 10164_2017_525_MOESM3_ESM.docx]

**Table S3**: Assessment of boldness of San Joaquin kit fox during trapping and handling

| **Eartag Number** | **Date** | | **Handler** |
| --- | --- | --- | --- |
| **Sex:** Male/Female | **Site:** Natural/Urban | | **Age:** Adult/Juvenile/Pup |
| **Vocalisations in trap** | | **Movement in Trap** | |
| 1. Warning bark | | 1. Running backwards and forwards | |
| Yes No | | Yes No | |
| 2. Growl/snarl | | 1. Crouching down and staying still | |
| Yes No | | Yes No | |
| 3. Scream (high pitched bark or yelp) | | 3. Moving away from handler | |
| Yes No | | Yes No | |
|  | | 4. Biting at cage | |
|  | | Yes No | |
|  | | 5. Entered into bag calmly | |
|  | | Yes No | |
|  | | 6. Defecated in trap | |
|  | | Yes No | |
| **Vocalisations in bag** | | **Behaviour in Bag and during handling** | |
| 1. Warning bark | | 1. Struggling | |
| Yes No | | Yes No | |
| 2. Growl/snarl | | 2. Remaining still | |
| Yes No | | Yes No | |
| 3. Scream (high pitched bark or yelp) | | 3. Defecated | |
| Yes No | | Yes No | |
|  | | 4. Biting at bag | |
|  | | Yes No | |
|  | | 5. Attempt to escape (e.g. eyes uncovered) | |
|  | | Yes No | |
