# Supplementary material for: Assessing personality in San Joaquin kit fox in situ: efficacy of field-based experimental methods and implications for conservation management
Source: J Ethol. 2017 Sep 12;36(1):23–33. doi: 10.1007/s10164-017-0525-9 (PMC5746588; doi:10.1007/s10164-017-0525-9)
Supplement: Supplementary file 4 — Supplementary material 4 (DOCX 18 kb) [file 10164_2017_525_MOESM4_ESM.docx]

**S4 - Evaluation of Methods used to Assess Personality in Wild San Joaquin kit fox**

**ENOT = Extended Novel Object Test**

**RNOT = Rapid Novel Object Test**

**T/H = Trap/Handling Test**

**ENOT**

1. Please rate the duration of time in weeks required to conduct ENOT where 1 is very little time and 5 is an extended period of time. Consider in your answer whether the Experiment can be fitted in alongside existing work.

1        2        3        4        5

### Please rate the amount of labour in terms of both number of people and man-hours required to collect data for ENOT, where 1 is a small labour requirement and 5 is a high labour requirement.

1        2        3        4        5

### Please rate the repeatability of ENOT, i.e. the ability to conduct multiple personality assessments on the SAME individual where 1 is low likelihood of repeatability and 5 is high likelihood of repeatability of the test.

1        2        3        4        5

### Please rate the quantity of data obtained from ENOT where 1 is low numbers of samples and 5 is high numbers of samples.

1        2        3        4        5

### Please rate the quality of data obtained from ENOT where 1 is data of limited quality and 5 is robust, thorough data.

1        2        3        4        5

### Please rate the expense associated with ENOT, where 1 is very low cost and five is very high cost. Please consider this in terms of both equipment and labour costs.

1        2        3        4        5

1. Please consider which age range of animals ENOT was suitable for. You can select more than one answer.

| Adult, juvenile and pup  Adult only  Juvenile only  Pup only  Adult and juvenile  Adult and pup  Juvenile and pup |
| --- |
|  |

1. Please consider if ENOT was biased in its design towards any one personality type.

| Yes, bold animals  Yes, shy animals  Yes, but with modification (e.g. hood-trapping) could obtain data from any personality type  No, worked as operated for any personality type |
| --- |

1. Please rate the potential or likelihood for failure with the ENOT data collection method in the field, where 1 is not very likely and 5 is very likely.

1        2        3        4        5

**RNOT**

1. Please rate the duration of time in weeks required to conduct RNOT where 1 is very little time and 5 is an extended period of time. Consider in your answer whether the Experiment can be fitted in alongside existing work.

1        2        3        4        5

### Please rate the amount of labour in terms of both number of people and man-hours required to collect data for RNOT, where 1 is a small labour requirement and 5 is a high labour requirement.

1        2        3        4        5

### Please rate the repeatability of RNOT, i.e. the ability to conduct multiple personality assessments on the SAME individual where 1 is low likelihood of repeatability and 5 is high likelihood of repeatability of the test.

1        2        3        4        5

### Please rate the quantity of data obtained from RNOT where 1 is low numbers of samples and 5 is high numbers of samples.

1        2        3        4        5

### Please rate the quality of data obtained from RNOT where 1 is data of limited quality and 5 is robust, thorough data.

1        2        3        4        5

### Please rate the expense associated with RNOT, where 1 is very low cost and five is very high cost. Please consider this in terms of both equipment and labour costs.

1        2        3        4        5

1. Please consider which age range of animals RNOT was suitable for. You can select more than one answer.

| Adult, juvenile and pup  Adult only  Juvenile only  Pup only  Adult and juvenile  Adult and pup  Juvenile and pup |
| --- |
|  |

1. Please consider if RNOT was biased in its design towards any one personality type.

| Yes, bold animals  Yes, shy animals  Yes, but with modification (e.g. hood-trapping) could obtain data from any personality type  No, worked as operated for any personality type |
| --- |

1. Please rate the potential or likelihood for failure with the RNOT data collection method in the field, where 1 is not very likely and 5 is very likely.

1        2        3        4        5

**Trap/Handling Test**

1. Please rate the duration of time in weeks required to conduct the Trap/Handling boldness assessment where 1 is very little time and 5 is an extended period of time. Consider in your answer whether the Experiment can be fitted in alongside existing work.

1        2        3        4        5

### Please rate the amount of labour in terms of both number of people and man-hours required to collect data for the Trap/Handling boldness assessment, where 1 is a small labour requirement and 5 is a high labour requirement.

1        2        3        4        5

### Please rate the repeatability of Trap/Handling boldness assessment, i.e. the ability to conduct multiple personality assessments on the SAME individual where 1 is low likelihood of repeatability and 5 is high likelihood of repeatability of the test.

1        2        3        4        5

### Please rate the quantity of data obtained from the Trap/Handling boldness assessment where 1 is low numbers of samples and 5 is high numbers of samples.

1        2        3        4        5

### Please rate the quality of data obtained from the Trap/Handling boldness assessment where 1 is data of limited quality and 5 is robust, thorough data.

1        2        3        4        5

### Please rate the expense associated with the Trap/Handling boldness assessment, where 1 is very low cost and five is very high cost. Please consider this in terms of both equipment and labour costs.

1        2        3        4        5

1. Please consider which age range of animals the Trap/Handling boldness assessment was suitable for. You can select more than one answer.

| Adult, juvenile and pup  Adult only  Juvenile only  Pup only  Adult and juvenile  Adult and pup  Juvenile and pup |
| --- |
|  |

1. Please consider if the Trap/Handling boldness assessment was biased in its design towards any one personality type.

| Yes, bold animals  Yes, shy animals  Yes, but with modification (e.g. hood-trapping) could obtain data from any personality type  No, worked as operated for any personality type |
| --- |

1. Please rate the potential or likelihood for failure with the Trap/Handling boldness assessment data collection method in the field, where 1 is not very likely and 5 is very likely.

1        2        3        4        5
