# Supplementary material for: Assessing personality in San Joaquin kit fox in situ: efficacy of field-based experimental methods and implications for conservation management
Source: J Ethol. 2017 Sep 12;36(1):23–33. doi: 10.1007/s10164-017-0525-9 (PMC5746588; doi:10.1007/s10164-017-0525-9)
Supplement: Supplementary file 5 — Supplementary material 5 (DOCX 22 kb) [file 10164_2017_525_MOESM5_ESM.docx]

a)

b)

c)

**Figure S5 a – c**: Plot of individual boldness scores obtained from the a) ENOT (n = 24), b) RNOT (n = 27) and c) TH boldness tests (n= 87)
