# Supplementary material for: Assessing personality in San Joaquin kit fox in situ: efficacy of field-based experimental methods and implications for conservation management
Source: J Ethol. 2017 Sep 12;36(1):23–33. doi: 10.1007/s10164-017-0525-9 (PMC5746588; doi:10.1007/s10164-017-0525-9)
Supplement: Supplementary file 6 — Supplementary material 6 (DOCX 23 kb) [file 10164_2017_525_MOESM6_ESM.docx]

Figure S1a-c **Assessment of efficacy by field researchers of boldness tests: Extended Novel Object Test, Rapid Novel Object Test and Trap – Handling test conducted on San Joaquin kit fox** (***Vulpes macrotis mutica***) **in Bakersfield, California.** (Duration, 1 = very little time & 5 = extensive; Labour requirements, 1=low, 5=high; Ease of Repeatability, 1 = hard, 5 = easy; Data Quantity, 1=little, 5=extensive; Data Quality, 1=poor, 5=robust; Expense, 1=low, 5= high; Likelihood of Failure, 1=low, 5 = high).
